# The Incidence of Mosaicism for Individual Chromosome in Human Blastocysts Is Correlated With Chromosome Length

**DOI:** 10.3389/fgene.2020.565348

**Published:** 2021-01-06

**Authors:** Tzu-Hsuan Chuang, Ya-Ping Chang, Meng-Ju Lee, Huai-Ling Wang, Hsing-Hua Lai, Shee-Uan Chen

**Affiliations:** ^1^Stork Fertility Center, Stork Ladies Clinic, Hsinchu City, Taiwan; ^2^Department of Obstetrics and Gynecology, National Taiwan University Hospital and College of Medicine, Taipei City, Taiwan

**Keywords:** mosaicism, chromosome length, next-generation sequencing, preimplantation genetic testing for aneuploidy, mitotic aberration

## Abstract

Mosaicism, known as partial aneuploidies, mostly originates from mitotic errors during the post-zygotic stage; it consists of different cell lineages within a human embryo. The incidence of mosaicism has not been shown to correlate with maternal age, and its correlation with individual chromosome characteristics has not been well investigated. In this study, the results of preimplantation genetic testing for aneuploidy (PGT-A) derived from 4,036 blastocysts (930 IVF couples) were collected from 2015 to 2017. Via next-generation sequencing for comprehensive chromosome screening, embryo ploidy was identified as aneuploid, mosaic, and euploid. Total mosaicism was classified into two categories: “mosaic euploid/aneuploidy” (with mosaic aneuploidy between 20 and 80%) and “mosaic and aneuploidy” (a uniformly abnormal embryo superimposed with mosaic aneuploidies). Frequency of mosaicism was analyzed according to the function of chromosomal lengths, which divides involved chromosomes into three groups: group A (156–249 Mb), group B (102–145 Mb), and group C (51–90 Mb). The results show that the aneuploidy was more frequent in group C than in group A and group B (A: 23.7%, B: 35.1, 41.2%, *p* < 0.0001), while the mosaicism was more frequent in group A and group B than in group C [(Mosaic euploid/aneuploid) A: 14.6%, B: 12.4%, C: 9.9%, *p* < 0.0001; (mosaic and aneuploid) A: 21.3%, B: 22.9%, C: 18.9%, *p* < 0.0001; (Total mosaicism) A: 35.9%, B: 35.3%, C: 28.8%, *p* < 0.0001]. The significantly higher frequency of aneuploidy was on the shorter chromosome (< 90 Mb), and that of mosaicism was on the longer chromosomes (> 100 Mb). The length association did not reach significance in the patients with advanced age (≥ 36 years), and of the chromosome-specific mosaicism rate, the highest prevalence was on chromosome 14 (5.8%), 1 (5.7%), and 9 (5.6%). Although the length association was observed via group comparison, there may be affecting mechanisms other than chromosomes length. Eventually, twenty patients with mosaic embryo cryotransfers resulted in six live births. No significant correlation was observed between the transfer outcomes and chromosome length; however, the analysis was limited by small sample size.

## Introduction

Meiotically derived chromosome aneuploidy (abbreviated as CA) occurs in both natural conception and *in vitro* fertilization (IVF), and its prevalence increases with maternal age. Aneuploid chromosome during meiosis originated from segregation error and is explained according by the two-hit model: first, formation of susceptible crossover structures; second, compromise of age-dependent decline in chromosome cohesion. Sakakibara et al. (2015) found that 80% of CA is attributed to the premature bivalence into univalence of chromosome segregation during meiosis I (MI) and could result in an imbalanced number of chromosomes. Clinical studies have shown that in cases of natural conception, about 0.3% of newborns having aneuploidy with the most common being trisomy 13, trisomy 18, trisomy 21, and sex chromosome aneuploidy. The incidence of CA in stillbirths is about 4%, and 35% in spontaneous miscarriage (Hassold and Hunt, 2001; Nagaoka et al., 2012). Of the CA in patients undergoing IVF, it was identified in preimplantation embryos at different culture stages (Fragouli et al., 2011; Taylor et al., 2014). Previous publications showed that embryos generated via strong stimulation protocols, or under certain culture conditions may also experience an increased prevalence of CA (Munne et al., 1997; Kline et al., 2011). Thus, CA was shown to be the primary cause of implantation failure in patients with IVF (Baart et al., 2006).

Embryonic mosaicism is defined as the occurrence of two or more cell populations with different karyotypes within an individual embryo. It includes a mixture of diploid-aneuploid cells exhibiting partially gain or loss of chromosomal ploidy (Fragouli et al., 2017; Lal et al., 2020). Euploid/aneuploid mosaicism mostly originates from mitotic errors during the post-zygotic stage. Unlike the aneuploidies of meiotic origin, euploid/aneuploid mosaicism is less associated with maternal age (Taylor et al., 2014). Previous studies have suggested that different mechanisms lead to mitotic aneuploidy: non-disjunction, anaphase lagging, endo-replication/deletion, premature cell division, and trisomy rescue (Taylor et al., 2014). Many studies have shown that mosaicism is commonly produced in 15–90% of cleavage-stage embryos and around 10–30% of blastocysts (Fragouli et al., 2011; Spinella et al., 2018). The concordance between trophectoderm (TE) and inner-cell mass (ICM), the involved chromosomes, and the percentage of aneuploidy in mosaic embryos may determine pregnancy outcomes and live births (Capalbo et al., 2017).

Recent reports showed that transfer of mosaic embryo resulted in poorer clinical outcomes than patients receiving euploid embryos (Victor et al., 2019). Fragouli et al. (2017) reported that both the implantation and ongoing pregnancy rates of mosaic embryo transfers were significantly lower than euploid embryo transfers; and the miscarriage rate was significantly higher. Although the overall outcomes of mosaic embryo transfer were compromised, they can still result in healthy newborns (Greco et al., 2015). Spinella et al. (2018) demonstrated that the outcomes of transfer of mosaic embryos with low aneuploidy (< 50%) were similar to those of euploid embryos. However, transfer of mosaic embryos with high aneuploidy (≥ 50%) resulted in a significantly lower pregnancy rates.

Preimplantation genetic testing for aneuploidy (PGT-A) is a widely used technology to identify chromosomally normal embryos before transfer, thereby avoiding the transfer of aneuploid embryos (Chen et al., 2017; Poli et al., 2019). It has been reported to increase pregnancy rate and reduce miscarriage rate per embryo transfer (Geraedts and Sermon, 2016). Advances in next-generation sequencing (NGS) in application of PGT-A also result in higher sensitivity, higher throughput, reduced time for testing, and lower costs (Wells et al., 2014). The combination of PGT-A/NGS can detect segmental aneuploidies and mosaicism with low-rate aneuploidy (Vera-Rodríguez et al., 2016; Lai et al., 2017; Hu et al., 2019). Because PGT-A/NGS has a robust capacity to detect chromosomal mosaicism in embryos, the application of embryo transfer priority based on the aneuploid percentage may improve reproductive outcomes (Maxwell et al., 2016).

Pylyp et al. (2018) showed that the chromosomes involved are linked to the pregnancy outcomes. Most aneuploidies are found in pregnancy loss or abnormal live births (e.g., chromosomes 8, 13, 16, 18, 20, 21, and 22) (Driscoll and Gross, 2009; Maxwell et al., 2016; Fragouli et al., 2017). Such aneuploidies are also frequently observed in preimplantation embryos with PGT-A (Nakhuda et al., 2018). In a study involving 15,169 embryo biopsies, aneuploidies involving chromosomes 13, 15, 16, 18, 19, 21, and 22 were found to be the most common in PGT-A testing (Franasiak et al., 2014). Furthermore, aneuploidies in sex chromosomes are also prevalent in both preimplantation and prenatal testing, including Turner syndrome (45, X), Klinefelter’s syndrome (47, XXY), or 47, XYY syndrome (Maxwell et al., 2016). Although particular chromosomes with higher frequencies of meiotic aneuploidy in preimplantation embryos have been investigated (Driscoll and Gross, 2009; Maxwell et al., 2016; Fragouli et al., 2017), the specific chromosomes that exhibit higher frequencies of mosaicism remain unclear. Because mosaic embryos could result in viable pregnancies, the prevalence and effects of mosaicism involving individual chromosomes is important. The purpose of this study was to investigate the mosaicism on individual chromosome distribution and associated chromosomal characteristics.

## Materials and Methods

### Study Subjects, Embryo Culture and Biopsy, Embryo Cryopreservation

The study was approved by the Ethics Reviewing Committee of National Taiwan University Hospital. The retrospective cohort dataset was collected from February 2015 through December 2017 at a private fertility center (Hsinchu, Taiwan). All the involved patients underwent personalized stimulation protocols for the IVF/PGT-A program (Wang et al., 2016; Hwang et al., 2018). The cohort included 698 couples with IVF using their own oocytes and 232 couples with a donated-oocyte program. Written informed consent was obtained from the individuals for the publication of any potentially identifiable images or data included in this article. A laser was used on the zona pellucida of derived embryos for assisted hatching on day 4 of culturing; the available blastocysts were then biopsied with approximately 5–10 TE cells on days 5 or 6 using mechanical shearing force between the biopsy and the holding pipettes (Origio, Måløv, Denmark). Only when both the ICM and TE reached B grading or above according to the Gardner and Schoolcraft system (Gardner and Schoolcraft, 1999) was the blastocyst biopsied and then cryopreserved. The biopsied specimens were washed twice in sterile 1X phosphate-buffered saline (PBS) solution (Cell Signaling Technologies, Danvers, MA, United States) containing 1% (w/v) polyvinylpyrrolidone (PVP; Sigma, St. Louis, MO, United States). Then, the biopsied specimens were gently transferred into 250 μl-tubes with 2.5 μl of PBS/PVP solution and stored at -20°C for analysis of NGS. The biopsied blastocysts were cryopreserved with a vitrification protocol (Cryotop^®^, Kitazato, Tokyo, Japan), and would be thawed if the couples would like to transfer.

### Cell Lysis, Whole-Genome Amplification, and Product Quantification

The Sureplex amplification system (Sureplex; Illumina, San Diego, CA, United States) was used for DNA extraction and amplification in the TE biopsies. A master mix, including cell extraction buffer, extraction enzyme dilution buffer, and extraction enzyme, was used to lyse the cells. Next, the released DNAs were fragmented and initially pre-amplified using Sureplex pre-amplification cocktail, which included Sureplex pre-amplification buffer and pre-amplification enzyme. Finally, the pre-amplified products were amplified using a Sureplex amplification cocktail containing amplification buffer, amplification enzyme, and nuclease-free water. A Qubit dsDNA HS (high-sensitivity) Assay Kit (Qubit^®^; Life Technologies, Waltham, MA, United States) was used to quantify the concentration of 10X diluted whole-genome-amplified products using a Qubit fluorometer (Qubit^®^; Life Technologies). Following the suggestions of manufacturer (Illumina), the products were diluted to 0.2 ng/μl for the following library preparation.

### NGS Library Preparation and Analysis

The Sureplex amplified dsDNA products (0.2 ng/μl) were prepared for the Veriseq library via the following steps: first, the dsDNA products were fragmented and then tagged with dual index primers using limited thermal cycles; next, the fragmented and tagged products were purified with AMPure beads via size selection (AMPure XP beads, Beckman Coulter, Pasadena, CA, United States) and normalized to equal concentration in individual sample; then, the final products were pooled to be sequenced using a Veriseq PGS kit (v.3) on a Miseq System (Illumina). Finally, we conducted copy number analysis using BlueFuse Multi Software (Illumina). The samples were distinguished if a median chromosomal copy number deviated from the default line; additional details of the aneuploidy calling regarding determinations of copy number variation were based on work by Fiorentino et al. (2014) and manufacturer’s instructions (Illumina, a technical guide to aneuploidy calling with Veriseq PGS).

### Mosaicism Validation and Determination

The conclusion of PGT-A was determined by the aneuploid percentage, which was adjusted by a validation curve derived from an aneuploid/diploid cells mixing experiment (Chuang et al., 2018). We choose the cut-off values of 20–80% of mosaicism in this study, based on both the 2016 PGDIS (Preimplantation Genetic Diagnosis International Society) position statement, as well as the result of the previous mixing experiment for internal validation on the Veriseq PGS system (Chuang et al., 2018). Aneuploid was defined as an aneuploid percentage above 80%; euploid was defined as an aneuploid percentage below 20%. According to Munné et al. (2016), total mosaicism was classified into two categories: mosaic euploid/aneuploid (as an embryo with so-called euploid/aneuploid mosaicism, here with an aneuploid percentage between 20 and 80%), mosaic and aneuploid (a uniformly abnormal embryo containing cells with mosaic aneuploidies, so a meiotic abnormality superimposed with post-zygotic mitotic abnormalities).

### Chromosome Classification

Chromosome grouping is a simplified division modified from the classification of chromosome length in Denver’s system (Supplementary Table 1). It synchronizes the number as eight chromosomes in each group, and thus the equal number of chromosome among three groups makes the following analysis of mosaicism frequency comparable: group A (156–249 Mb, including chromosomes 1, 2, 3, 4, 5, 6, 7, X) mainly referred to Denver’s class A–C, group B (102–145 Mb, including chromosomes 8, 9, 10, 11, 12, 13, 14, 15) mainly referred to Denver’s class C–D, and group C (51–90 Mb, including chromosomes 16, 17, 18, 19, 20, 21, 22, Y) referred to Denver’s class E–G.

### Analysis of Mosaicism Frequency in Relation With Chromosomal Length

According to the previous scientific articles, the chromosome mis-segregation rate showed size association with the chromosome lengths through the animal and human cell line models (Drpic et al., 2018; Dumont et al., 2019), and it has not been analyzed in the clinical data of PGT-A. Therefore, we investigated the frequency of mosaicism as well as aneuploidy for individual chromosome in relation with three groups as a function of chromosome length for all embryos screened by PGT-A. Percentages of mosaicism are calculated on the number of chromosomes involved total mosaicism events. The couples with PGT-A included 698 IVF couples using their own oocytes (75.1%) for various indications with an average female age of 36.5 years and 232 couples using donated oocytes (24.9%) with an average donor age of 24.2 years. Because of the wide span of maternal age in the study cohort, we further analyzed the frequencies of mosaicism and aneuploidy in relation with chromosomal length based on different age spans (≥ 36 and ≤ 32 years) (Royal College of Obstetricians and Gynecologists Statement on later maternal age, 2011; Taiwanese Databank of the Gender Equality Committee, 2015–2017).

### Transfer of Mosaic Euploid/Aneuploid Embryos and Pregnancy Follow-Up

For the mosaic embryos, the fertility specialists, and consultants would discuss the risks of discarding a competent embryo vs. transferring an embryo with a lower implantation potential and possible adverse obstetrical and neonatal outcomes with the couples based on the 2016 PGDIS position statement. Recommendations regarding prenatal screening and diagnoses were made. The couples decided whether to transfer mosaic embryos. The clinical pregnancy rate (CPR) was defined as the number of transfer cycle with a detected gestational sac(s) observed via ultrasound divided by the total number of transfer cycles. The implantation rate (IR) was defined as the number of detected gestational sac(s) per transferred embryo. The live birth rate was defined as the number of transfer cycles with successful live births divided by the total number of transfer cycles.

### Statistical Analysis

The count data are presented as percentages, and the continuous data are presented as averages with standard deviations (SD). The chi-square test was used to compare the frequencies among groups, and the chi-square for trend was used to evaluate correlation between chromosome lengths and frequencies. A significant difference was defined as a *p*-value below 0.05. All of the analyses were conducted using scientific GraphPad software (Prism; GraphPad Software, La Jolla, CA, United States).

## Results

### Characteristics of Patients With PGT-A

The patient data are listed in Table 1. The studied cohort consisted of 930 couples with IVF, including 698 couples using their own oocytes (mean age: 36.5 years) and 232 couples using donated oocytes (mean age of donors: 24.2 years). The indications of PGT-A included donated oocyte recipient deciding single embryo transfer (24.9%), advanced maternal age (≥ 36 years) (46.5%), male factor (4.1%), and repeated implantation failure (24.5%).

**TABLE 1:** Characteristics of patients with PGT-A.

	IVF using own oocytes	IVF using donated oocytes
Number of patients	698	232
Mean female age, donor age (range) (years)	36.5 (19–52)	24.2 (20–32)
Baseline AMH^a^ (ng/ml)	4.0 ± 0.1	7.0 ± 0.3
Antral follicle count	9.6 ± 0.2	14.2 ± 0.4
Indications		
Donated oocyte recipients for SET^b^ (%)	232 (24.9%)	
Advanced maternal age (> 36 years)^c^ (%)	432 (46.5%)	
Male factor (%)	38 (4.1%)	
Repeated implantation failure (%)	228 (24.5%)	

**^*a*^AMH, anti-mullerian hormone. ^*b*^Donated oocyte recipients deciding single embryo transfer (SET) and requesting PGT-A to select the best embryo for SET. ^*c*^Women aged 36 years and above.**

### Results of Embryo Screened by PGT-A

There were 4,036 embryos for TE biopsy, and 70 biopsies failed to be amplified (1.7%, 70/4,036). Therefore, 3,966 embryos were screened by PGT-A/NGS. Of these samples, 1,709 embryos were euploid (43.1%, 1,709/3,966), and 1,044 embryos were aneuploid (26.3%, 1,044/3,966). A total of 548 mosaic euploid/aneuploid (13.8%, 548/3,966) and 626 mosaic and aneuploid (15.8%, 626/3,966), were identified by NGS. The details of PGT-A results were shown in Table 2.

**TABLE 2:** Results of embryos screened by PGT-A.

Number of patients with PGT-A	930
Number of embryos biopsied	4,036
Number of embryos screened by PGT-A	3,966
Number of embryos that failed to be amplified	70(1.7%)
Euploid^a^ (%)	1,709(43.1%)
Aneuploid^b^ (%)	1,044(26.3%)
Mosaic euploid/aneuploid^c^ (%)	548(13.8%)
Single mosaic euploid/aneuploid (%)	393(71.7%)
Double mosaic euploid/aneuploid (%)	75(13.7%)
Multiple^e^ mosaic euploid/aneuploid (%)	80(14.6%)
Mosaic and aneuploid^d^ (%)	626(15.8%)
Single mosaic and aneuploid (%)	279(44.5%)
Double mosaic and aneuploid (%)	167(26.7%)
Multiple^e^ mosaic and aneuploid (%)	180(28.8%)
Inconclusive^f^ (%)	39(1.0%)

**^*a*^A euploid embryo is defined as with under 20% aneuploidy. ^*b*^An aneuploid embryo is defined as with above 80% aneuploidy. ^*c*^A mosaic euploid/aneuploid embryo is defined as with between 20 and 80% aneuploidy. ^*d*^A mosaic and aneuploid embryo is defined as having both aneuploid (> 80%) and mosaic euploid/aneuploid (20–80%) signs. ^*e*^Multiple mosaicism is defined as more than two chromosomes with mosaicism. ^*f*^Due to inferior outcomes of quality control in the NGS analysis.**

### Higher Frequency of Mosaicism in Longer Chromosomes

**FIGURE 1:**
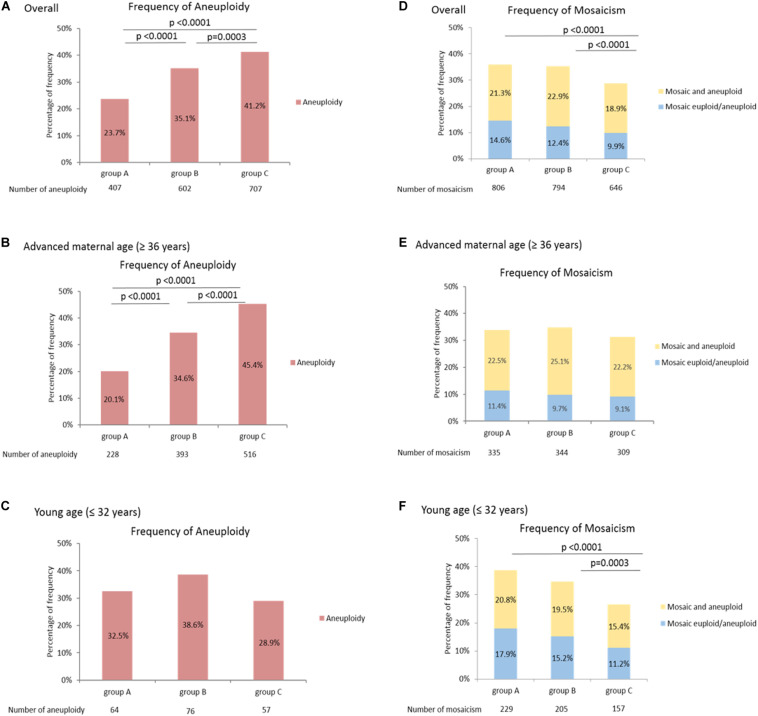
Higher frequency of mosaicism in longer chromosomes. The frequency of aneuploidy differed among the three groups as a function of chromosome length **(A-C)**. The frequency of aneuploidy was significantly higher within the shorter chromosomes in the overall patients **(A)** and patients with advanced maternal age (≥ 36 years) **(B)**, but the trend was not observed in the patients with young age (≤ 32 years) **(C)**. The frequency of mosaicism differed among the three groups as a function of chromosome length **(D-F)**. Both the frequencies of mosaic euploid/aneuploid and mosaic and aneuploid were significantly higher within the longer chromosomes in the overall patients **(D)** and the patients with young age (≤ 32 years) **(F)**, but the significance was not reached in the patients with advanced maternal age (≥ 36 years) **(E)**. The power of significance in the figures were shown by the *p*-values, and *p*≥ 0.05 is defined as without significance, which would not be labeled.

Figure 1 shows the frequencies of aneuploidy and mosaicisms in the three groups as a function of chromosome length. The frequency of aneuploidy was significantly higher in group C, which included shorter chromosomes (A: 23.7%, B: 35.1%, C: 41.2%, *p* < 0.0001), and the trend between the frequency of aneuploidy and chromosome length was significant in the overall patients (*p* < 0.0001) (Figure 1A). Moreover, the correlation between the frequency of aneuploidy and chromosome length was observed in the patients with advanced maternal age (≥ 36 years), but not in the patients with young age (≤ 32 years) (Figures 1B,C). Of the frequency of mosaicism, both the mosaic euploid/aneuploid and mosaic and aneuploid were significantly higher in the groups with longer chromosomes [(mosaic euploid/aneuploid) A: 14.6%, B: 12.4%, C: 9.9%, *p* < 0.0001; (mosaic and aneuploid) A: 21.3%, B: 22.9%, C: 18.9%, *p* < 0.0001; (Total mosaicism) A: 35.9%, B: 35.3%, C: 28.8%, *p* < 0.0001]. The trend of increasing mosaicism with longer chromosome is observed between group A and group C, group B and group C, but not between group A and group B (Figure 1D). Both the frequencies of mosaic euploid/aneuploid and mosaic and aneuploid were significantly higher within the longer chromosomes in the overall patients and in the patients with young age (≤ 32 years) (Figure 1F), but the significance of chromosome size association was not reached in the patients with advanced maternal age (≥ 36 years) that would be due to a lack of power (Figure 1E). Unlike aneuploidy, which tended to occur in the shorter chromosomes, mosaicism frequently occurs in the longer chromosomes. However, the prevalence of aneuploidy and mosaicism in the older and younger women displayed different distribution. Details of the above mosaicism calculations by groups were displayed in Supplementary Table 2.

### Mosaicism and Aneuploidy Across Individual Chromosomes

**FIGURE 2:**
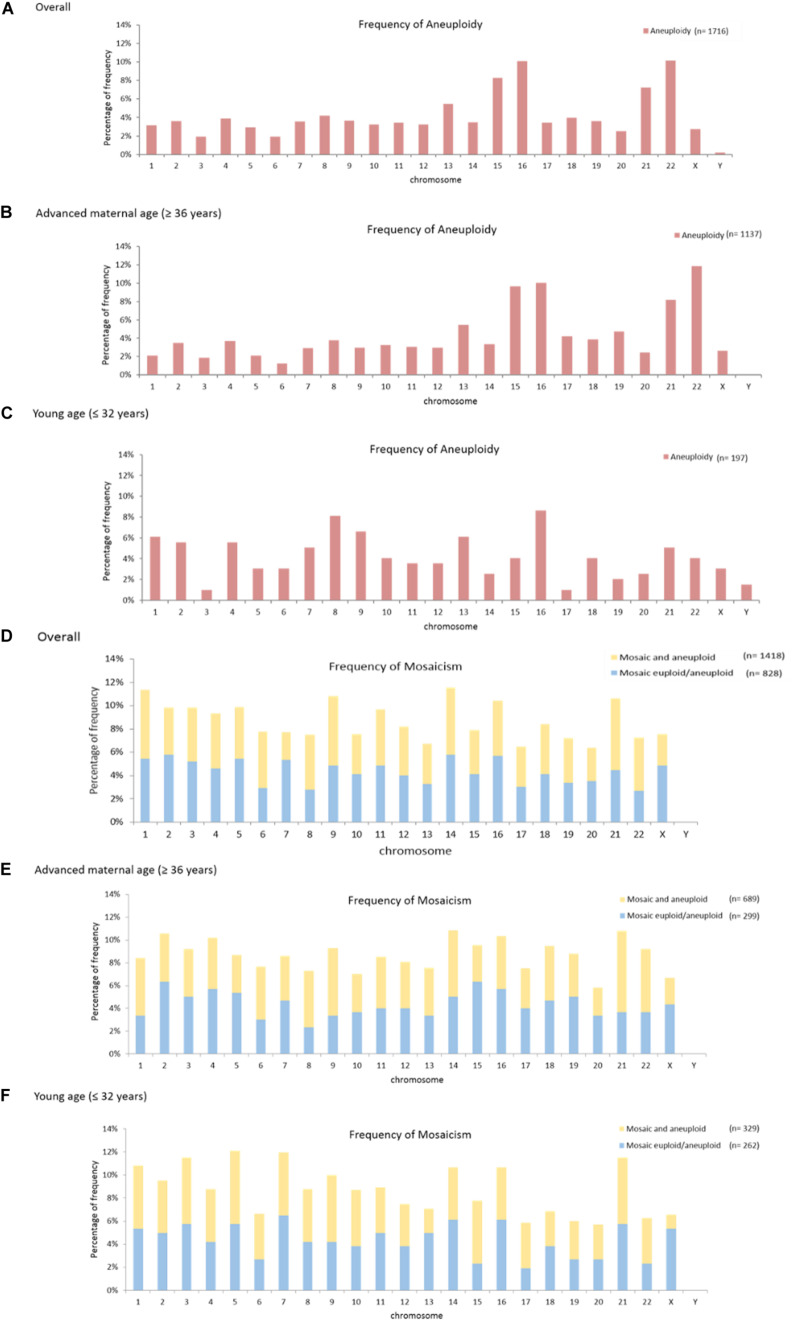
Mosaicism and aneuploidy across individual chromosomes. The frequency of aneuploidy across individual chromosomes was shown **(A-C)**. The highest frequencies in the overall patients occurred on chromosome 22, 16, and 15 **(A)**. The highest frequencies in the patients with advanced maternal age (≥ 36 years) occurred on chromosomes 22, 16, and 15 **(B)**. The highest frequencies in the patients with young age (≤ 32 years) occurred on chromosomes 16, 8 and 9 **(C)**. The frequency of mosaicism, including both the mosaic euploid/aneuploid and mosaic and aneuploid, across individual chromosomes was shown **(D-F)**. The highest frequency of mosaicism in the overall patients occurred on chromosomes 14, 1 and 9 **(D)**. The highest frequency of mosaicism in the patients with advanced maternal age (≥ 36 years) occurred on chromosomes 21,14 and 9 **(E)**. The highest frequency of mosaicism in the patients with young age (≤ 32 years) occurred on chromosomes 5, 7, 3, and 21 **(F)**.

Figure 2 shows the frequencies of aneuploidy and mosaicisms across individual chromosomes. Aneuploidy often occurred on chromosomes 22 (10.1%), 16 (10.1%), and 15 (8.3%) (Figure 2A). Aneuploidy on chromosome 16 shows higher frequency in both the patients with advanced maternal age (10.0%) and with young age (8.6%) (Figures 2B,C). Of mosaicism involving individual chromosome, mosaic euploid/aneuploid most often occurred on chromosomes 2 (5.8%), 14 (5.8%), and 16 (5.7%); and mosaic and aneuploid most often involved chromosomes 21 (6.1%), 9 (6.0%), and 1 (5.9%). In total the mosaicism involved chromosomes 14 (5.8%), 1 (5.7%), and 9 (5.6%). Chromosomes with highest frequency of mosaicism in the overall patients was concordant with those in the patients with advanced maternal age (Figures 2D,E), but not in the patients with young age (Figure 2F). Details of the above mosaicism calculations per chromosome were displayed in Supplementary Table 3.

### Clinical Outcomes of Mosaic Embryo Transfer

**FIGURE 3:**
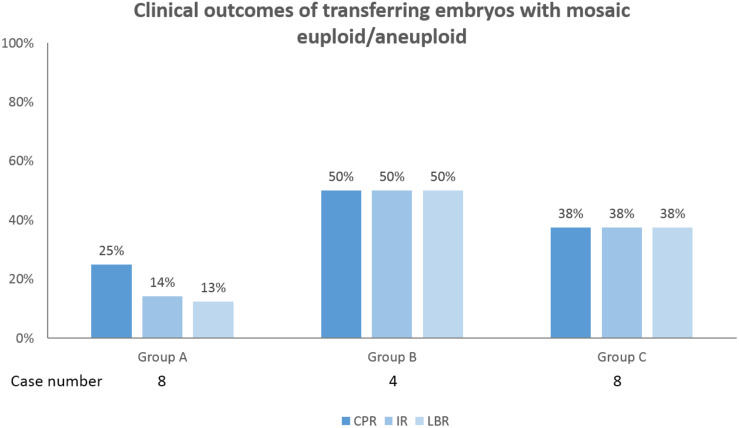
Clinical outcomes of transferring embryos with mosaic euploid/aneuploid in 20 patients were shown. Among the three groups as a function of chromosome length, the clinical pregnancy rate (CPR), implantation rate (IR), ongoing pregnancy rate (OPR), and live birth rate (LBR) were not significantly correlated with chromosome length. The power of significance in the figures were shown by the *p*-values, and *p*≥ 0.05 is defined as without significance, which would not be labeled.

Twenty patients underwent cryotransfers with mosaic euploid/aneuploid embryos (Table 3). Seven patients had positive pregnancy tests. Thirteen gestational sacs and fetal heartbeats were detected. One patient lost her pregnancy in the first trimester. Six patients underwent prenatal tests for chromosome states: two with first-trimester Down’s screening tests, three with non-invasive prenatal testing, and one with amniocentesis. All of the results were normal. Eventually, 11 healthy babies were delivered. The overall clinical outcomes were as follows: clinical pregnancy rate: 35.0% (7/20), implantation rate: 31.6% (12/38), and live birth rate: 30.0% (6/20). Figure 3 illustrates the clinical outcomes against the three groups as a function of chromosome length. The clinical pregnancy rate, implantation rate, ongoing pregnancy rate, and live birth rate did not exhibit significant size association.

**TABLE 3:** Clinical outcomes of transferring embryos with mosaic euploid/aneuploidy.

Patient no.	Age (years)	Oocyte source	Embryo no.	Ploidy	Chromosome length^a^	Prenatal chromosome state	Clinical outcome
1	36	Self	2	ch.11 (−28%)	B	Normal	Twins
2	25	Self	2	ch.5 (+45%)	A	−	No pregnancy
3	44	Self	2	ch.19 (−41%)	C	Normal	Twins
4	36	Self	2	ch.2 (+30%)	A	−	Spontaneous miscarriage
5	36	Self	1	ch.3 (+27%)	A	−	No pregnancy
6	46	Donated	2	ch.19 (−50%)	C	−	No pregnancy
7	36	Self	2	ch.20 (+21%)	C	−	No pregnancy
8	36	Self	2	ch.7 (−42%)	A	−	No pregnancy
9	34	Self	2	ch.17 (−26%)	C	Normal	Twins
10	38	Self	2	ch.16 (+49%)	C	Normal	Twins
11	34	Self	1	ch.5 (−40%)	A	Normal	Singleton
12	40	Self	2	ch.11 (+33%)	B	−	No pregnancy
13	36	Self	2	ch.10 (−35%)	B	Normal	Twins
14	42	Donated	2	ch.9 (−48%)	B	−	No pregnancy
15	42	Self	2	ch.4 (−40%)	A	−	No pregnancy
16	27	Donated	2	ch.18 (+40%)	C	−	No pregnancy
17	38	Self	2	ch.21 (+40%)	C	−	No pregnancy
18	46	Donated	2	ch.7 (+22%)	A	−	No pregnancy
19	31	self	2	ch.1 (−27%)	A	−	No pregnancy
20	44	Donated	2	ch.Y (−38%)	C	−	No pregnancy

**^*a*^Group A includes chromosomes with lengths of 156–249 Mb, including ch.1, 2, 3, 4, 5, 6, 7, X; group B includes chromosomes with lengths of 102–145 Mb, including ch.8, 9, 10, 11, 12, 13, 14, 15; group C includes chromosomes with lengths of 51–90 Mb, including ch.16, 17, 18, 19, 20, 21, 22, Y.**

## Discussion

Our data demonstrated that mosaicism as identified during the analysis of the trophectoderm in the blastocyst stage more frequently involves longer chromosomes regardless of the female age. Furthermore, the clinical outcomes of transferring mosaic euploid/aneuploid embryo were not correlated with the lengths of the chromosome involved. However, our sample size was too small to draw definitive conclusions and further studies involving larger cohorts are necessary to confirm these findings.

To date, most studies have focused on the distribution of mosaicism across individual chromosomes rather than analyzing the correlation of mosaicism and specific chromosomal characteristics in human embryos. McCoy et al. (2015) observed no increased mitotic error rates in larger chromosomes in day 5-blastocysts. We showed that the frequency of mosaicism was significantly higher on chromosomes with longer lengths of 156–249 Mb compared with chromosomes with shorter lengths of 51–90 Mb; the frequency of mosaicism was also higher on chromosomes with median lengths of 102–145 Mb than on chromosomes with shorter lengths of 51–90 Mb. The differences between our results and those of previous studies may be the results of two factors: first, the frequency of mosaicism was analyzed by three groups in this study to make the dataset simple as well as large enough to compare, while in the study of McCoy et al. (2015), it was analyzed by individual chromosomes. Second, both mosaic euploid/aneuploid and mosaic and aneuploid were evaluated in this study; McCoy et al. (2015) did not note which type(s) of mosaicism they studied. Nakhuda et al. (2018) who investigated only mosaic euploid/aneuploid reported that the most prevalent chromosome mosaicism involved chromosomes 21, 22, and 2, and most infrequently involved chromosomes 10, 12, and Y. However, in our study, the total mosaicism containing both mosaic euploid/aneuploid and mosaic and aneuploid was assessed and revealed that the chromosomes with the highest frequencies were 14, 1, and 9. In addition, Nakhuda et al. (2018) analyzed the mosaicism rate across individual chromosome dividing into whole and segmental chromosomes, respectively. We tried to apply possibly the same calculation to re-analyze our data and no concordance was found (data not shown). One of possible explanations for the discrepancy in the chromosome-specific rate of mosaicism may be the result of the definition and calling criteria of mosaicism in the different studies. Furthermore, although the frequency of mosaicism was significantly higher on chromosomes with longer lengths compared with chromosomes with shorter lengths through group comparison, some shorter chromosomes still showed higher mosaicism rate, including chromosome 16 and 21. There may be affecting mechanisms other than chromosomes length, and thus additional approaches to investigate the association of mosaicism frequency and individual chromosome structure were needed.

On the other hand, the distribution of chromosome aneuploidy displayed higher frequency in the shorter chromosomes (mainly in group C) of this study. The distribution pattern was quite similar with the previous articles of Franasiak et al. (2014) and Nakhuda et al. (2018). The most prevalent chromosome aneuploidy involved chromosome 15, 16, 21, and 22, in both the present and previous studies. The distribution of aneuploid frequency across individual chromosome displayed different patterns between the older (≥ 36 years) and younger (≤ 32 years) age spans. The non-linear relationship for meiotic aneuploidy and chromosome size with women’s age might relate to the recent description of different mechanisms for segregation error in young and old females by Gruhn et al. (2019). They found that whole-chromosome non-disjunction events are preferentially associated with increased aneuploidy in young females, whereas centromeric and more extensive cohesion loss preferred in old women.

The primary mechanisms of embryonic mosaicism were attributed to chromosome mis-segregation in mitosis. During the metaphase of mitosis, a cell establishes a bipolar spindle apparatus consisting of centrioles, microtubules, and replicated chromatids. Centromeres of individual chromosome play crucial docking sites for assembly of kinetochore, which is responsible for attachment of spindle microtubules. In a human somatic cell line (human retina pigmented epithelium, RPE-1), Dumont et al. (2019) discovered that inter-chromosomal heterogeneity of centromeric features influenced the chromosome segregation fidelity, and also showed the higher tendency of mis-segregation rate in larger chromosomes via measurement of centromere protein B (CENP-B) and kinetochore proteins. They found that small chromosomes with big centromeres displayed least mis-segregation rate; big chromosomes with small centromeres exhibited highest mis-segregation rate; and the mis-segregation rate of big chromosomes with big centromeres was in between. In an Indian muntjac fibroblast cell model, Drpic et al. (2018) found that chromosomes with larger kinetochores were biased to establish erroneous merotelic attachments and mis-segregated during anaphase. Kinetochore size is also a significant determinant of chromosome segregation fidelity. These mechanisms may provide possible reasons of the present results that the higher mis-segregation rate in mitosis happens in larger chromosomes in human blastocysts as well.

Besides the centromeric feature, other regulators involving mitotic modulation could affect the development of mosaic embryo. Vázquez-Diez et al. (2019) recently demonstrated that the modulation of chromosome segregation via spindle assembly checkpoint (SAC) and anaphase-promoting complex (APC/C) in the mammalian embryos that is very different from somatic cells. The averted signaling of SAC fails to impede anaphase onset when chromosome misalignment occurs in the early mouse morulae and blastocysts, and thus the chromosome error is retained in the progeny cells. They had observed that significantly increased amount of micronuclei observed in mouse morula or blastocyst stage. It implied that the embryos with aneuploid cells tried to isolate these unpaired or damaged chromosomes rejoining the principal nucleus (Vázquez-Diez et al., 2016; Vázquez-Diez and FitzHarris, 2018). In another mouse model, the elimination of abnormal ICM cells by apoptosis and limited proliferation of abnormal TE cells were seen in mosaic embryos (Bolton et al., 2016). In our study, no correlation was noted between clinical outcomes and affected chromosome lengths in the mosaic euploid/aneuploid embryo transfers. This could be driven by the above protecting mechanisms, which determine the different fate of varied aneuploid lineages. However, a sample size of mosaic embryo cryotransfer could lead an uninformative observation, and preimplantation chromosome aneuploidies are not absolutely preserved during human embryo development. Many studies have also reported that mosaic euploid/aneuploid embryo transfer in IVF can result in normal live births, suggesting that some mosaic embryos can self correct and possess developmental potential (Greco et al., 2015; Victor et al., 2019).

This study was limited by its retrospective nature. Nowadays, clinical detection of mosaicism is challenged in two regards: biological and technical. Of biological challenges, the distribution of cell lineages in a preimplantation embryo could not be even, and confined type of mosaicism occurred. It implies that the TE may not accurately reflect the genetic materials in the ICM (Chuang et al., 2018). Of the technical challenges, the number of cells in each TE biopsy was roughly 5–10, and the process of WGA in these cells might execute unevenly. Deviations in intermediate copy numbers in analytical software can originate from phenomena other than actual mosaicism, resulting in false-positive or false-negative outcomes during the analysis (Goodrich et al., 2016). Therefore, one should consider the effects of both the representativeness of a single TE biopsy and technical artifacts during comprehensive chromosome screening. Additionally, mixed population (self and donated oocytes) could make the biases derived from individual backgrounds. An appropriate background controlling in the study cohort and age stratification were needed in the future investigation.

## Conclusion

In conclusion, we have demonstrated that embryo mosaicisms tend to occur in longer chromosomes with lengths of at least above 100 Mb. This finding suggests a chromosome length-specific susceptibility to mitotic errors. Some mosaic embryos can result in live births, and no correlation was noted between the affected chromosome lengths and clinical outcomes. However, additional studies to determine the chromosomal characteristics in mosaic embryos are needed, since the underlying mechanism causing mosaicism is complex and there may be affecting mechanisms other than chromosomes length.

## Data Availability Statement

The original contributions presented in the study are included in the article/Supplementary Material, further inquiries can be directed to the corresponding author/s.

## Ethics Statement

The studies involving human participants were reviewed and approved by the Research Ethics Committee C, National Taiwan University Hospital. The ethics committee waived the requirement of written informed consent for participation. Written informed consent was obtained from the individuals for the publication of any potentially identifiable images or data included in this article.

## Author Contributions

T-HC and Y-PC designed the study concept and wrote the article. M-JL, H-LW, and H-HL recruited the patients. S-UC reviewed the article, revised the final version; and all the authors approved the submitted version.

## Conflict of Interest

The authors declare that the research was conducted in the absence of any commercial or financial relationships that could be construed as a potential conflict of interest.
