# Supplementary material for: The Incidence of Mosaicism for Individual Chromosome in Human Blastocysts Is Correlated With Chromosome Length
Source: Front Genet. 2021 Jan 6;11:565348. doi: 10.3389/fgene.2020.565348 (PMC7815765; doi:10.3389/fgene.2020.565348)
Supplement: Supplementary file 1 [file Data_Sheet_1.pdf]

Supplementary Table I Simplified system of division

Denver’s system *	Chromosome	Simplified division	Span of length
A	Ch.1-3	A	156-249 Mb
B	Ch.4-5	A	
C	Ch.6-12, X	A: Ch.6-7, X	
		B: Ch.8-12	102-145 Mb
D	Ch.13-15	B	
E	Ch.16-18	C	51-90 Mb
F	Ch.19-20	C	
G	Ch.21-22, Y	C	

* The relative position of centromere is not synchronized between the groups of this simplified system, since bin count reads for copy number variation were not calculated in para-centromere zone via Bluefuse Multi software.
