# Supplementary material for: The Incidence of Mosaicism for Individual Chromosome in Human Blastocysts Is Correlated With Chromosome Length
Source: Front Genet. 2021 Jan 6;11:565348. doi: 10.3389/fgene.2020.565348 (PMC7815765; doi:10.3389/fgene.2020.565348)
Supplement: Supplementary file 2 [file Data_Sheet_2.pdf]

Supplementary Table II Percentage of mosaicism frequency by groups

Group	A	B	C	Total	
Mosaic euploid/aneuploid	327	279	222	828	Chromosomes involved mosaic euploid/aneuploid events
Mosaic and aneuploid	479	515	424	1418	Chromosomes involved mosaic and aneuploid events
Total	806	794	646	2246	Chromosome involved total mosaicism events
	Chromosomes in A/B/C groups involved total mosaicism events				
Group	A	B	C	Total	
Mosaic euploid/aneuploid rate	14.6% (327/2246)	12.4% (279/2246)	9.9% (222/2246)		
Mosaic and aneuploid rate	21.3% (479/2246)	22.9% (515/2246)	18.9% (424/2246)		
Total mosaicism rate	35.9% (806/2246)	35.3% (794/2246)	28.8% (646/2246)	100%	
