# Supplementary material for: The Incidence of Mosaicism for Individual Chromosome in Human Blastocysts Is Correlated With Chromosome Length
Source: Front Genet. 2021 Jan 6;11:565348. doi: 10.3389/fgene.2020.565348 (PMC7815765; doi:10.3389/fgene.2020.565348)
Supplement: Supplementary file 3 [file Data_Sheet_3.pdf]

Supplementary Table III Percentage of mosaicism per chromosome

Chromosome	1	2	3	4	5	6	7	8	9	10	11	12	13	14	15	16	17	18	19	20	21	22	X	Y	Total
M E/A[a] events	45	48	43	38	45	24	44	23	40	34	40	33	27	48	34	47	25	34	28	29	37	22	40	0	828
M and A[b] events	84	57	66	67	63	69	34	67	85	49	69	60	49	82	54	67	49	61	54	41	87	65	39	0	1418
Total M[c] event	129	105	109	105	108	93	78	90	125	83	109	93	76	130	88	114	74	95	82	70	124	87	79	0	2246
M E/A rate (%)	5.4	5.8	5.2	4.6	5.4	2.9	5.3	2.8	4.8	4.1	4.8	4.0	3.3	5.8	4.1	5.7	3.0	4.1	3.4	3.5	4.5	2.7	4.8	0.0	
M and A rate (%)	5.9	4.0	4.7	4.7	4.4	4.9	2.4	4.7	6.0	3.5	4.9	4.2	3.5	5.8	3.8	4.7	3.5	4.3	3.8	2.9	6.1	4.6	2.8	0.0	
Total M rate (%)	5.7	4.7	4.9	4.7	4.8	4.1	3.5	4.0	5.6	3.7	4.9	4.1	3.4	5.8	3.9	5.1	3.3	4.2	3.7	3.1	5.5	3.9	3.5	0.0	

[a] M E/A, mosaic euploid/aneuploid

[b] M and A, mosaic and aneuploid

[c] Total M, total mosaicism
